# Community exercise programmes for chronic disease and the emerging role of physical activity for health officers in Ireland: a narrative review

**DOI:** 10.3389/fspor.2026.1960508

**Published:** 2026-10-21

**Authors:** Stephen McNally

**Affiliations:** Longford Sports Partnership/Longford County Council, County Longford Council, Longford, Ireland

**Keywords:** chronic disease, community exercise, exercise referral, health systems, Ireland, physical activity

## Abstract

**Background:**

Community-based exercise programmes are increasingly recognised as an important component of chronic disease management, supporting physical function, self-management, and long-term health outcomes. In Ireland, the Physical Activity for Health Officer (PAfHO) role has been introduced as a pilot initiative to strengthen pathways between healthcare services and community-based physical activity provision.

**Objective:**

To examine the evidence relating to community exercise programmes for chronic disease management and explore the emerging role of Physical Activity for Health Officers within the Irish health system. A narrative review of the literature was undertaken. Electronic databases (PubMed, Scopus, and Web of Science) and relevant grey literature sources were searched for contemporary evidence published from January 2020 to 2026. Literature relating to community exercise interventions, exercise referral and signposting pathways, integrated care models, and the PAfHO initiative in Ireland was reviewed and synthesised narratively. The final search date and screening totals are reported in the Methods and Results.

**Results:**

The literature indicates that community-based exercise programmes can improve physical activity participation, functional capacity and health-related quality of life among people living with chronic disease. Interventions incorporating personalised exercise, behavioural support and coordinated referral or transition pathways appear particularly promising. International evidence supports closer integration between healthcare and community physical activity provision. In contrast, evidence directly evaluating the Irish PAfHO model remains limited and is currently derived predominantly from policy, service-development and emerging implementation evidence. The model may facilitate referral and signposting pathways, access to community provision and cross-sector collaboration, but its effects on participant outcomes, healthcare utilisation and service efficiency require prospective evaluation.

**Conclusions:**

Community-based exercise represents a potentially scalable component of chronic disease management within integrated care systems. The emerging PAfHO model is consistent with international approaches emphasising coordinated transitions between healthcare and community physical activity provision. However, evidence supporting community exercise interventions is substantially more established than evidence evaluating the PAfHO role itself. Further research should determine whether the PAfHO model improves participant outcomes, access and continuity of physical activity participation, healthcare utilisation and cost-effectiveness.

## 1 Introduction

Chronic diseases are the leading contributors to morbidity, mortality, and healthcare utilisation globally. Physical inactivity is a major modifiable risk factor, contributing to disease progression, reduced functional capacity, and diminished quality of life. Despite established recommendations from the World Health Organization (WHO) advocating regular physical activity for adults, including those with chronic conditions, a substantial proportion of individuals remain insufficiently active, particularly those living with multimorbidity and disability (1). Bull et al. (1) recommend that adults engage in regular physical activity and minimise sedentary behaviour to improve health outcomes. This inactivity contributes not only to worsening health outcomes but also to increased healthcare demand and system-level pressures.

Healthcare systems have traditionally relied on episodic, clinician-led interventions, which are often insufficient to support sustained behaviour change or long-term condition management. While such models are effective in addressing acute or impairment-focused needs, they may fail to provide the continuity required for ongoing physical activity participation. Consequently, there has been a growing shift toward community-based exercise programmes and exercise referral pathways, which aim to extend care beyond clinical settings and support long-term self-management (2, 3). These programmes provide structured, accessible opportunities for individuals to engage in physical activity within their communities, often following discharge from formal rehabilitation services.

Emerging evidence suggests that the effectiveness of these programmes is influenced not only by the exercise intervention itself but also by the design of referral pathways, integration with healthcare services, and the presence of behavioural support mechanisms (3–5). Programmes that are personalised, socially supportive, and embedded within coordinated care pathways may be more likely to achieve sustained engagement, although the relative contribution of individual programme components remains uncertain (4, 6). This reflects a broader shift in health service delivery toward integrated, population-based models of care, where prevention, self-management, and community participation are prioritised alongside clinical treatment.

In Ireland, this shift is reflected in national policy, including the National Physical Activity Framework 2024–2040 and Sláintecare, both of which emphasise prevention, community-based care, and integrated service delivery (40). These policy frameworks highlight the importance of creating sustainable pathways that enable individuals to transition from healthcare services to long-term physical activity within their communities. However, translating policy into practice requires operational models that can effectively link clinical services with community provision.

Within the Irish health system, people living with chronic conditions may receive care across general practice, HSE primary and community care services, and hospital-based specialist or rehabilitation services. The PAfHO role is positioned at the interface between these healthcare services and community-based physical activity provision, rather than as a substitute for clinical diagnosis, treatment or rehabilitation.

Within this context, the Physical Activity for Health Officer (PAfHO) pilot has been introduced as a cross-sectoral initiative aimed at strengthening pathways between the Department of Health/Health Service Executive (HSE), Sport Ireland and local authorities/sports partnerships. The PAfHO role is designed to facilitate signposting, coordination, and programme development, with a particular focus on individuals living with chronic conditions and those who are least active. As such, it represents a novel service model positioned at the interface between healthcare and community systems, with potential to address gaps in current care pathways. Despite its policy relevance and practical importance, there is currently limited peer-reviewed evidence evaluating the PAfHO model or examining its role within the broader context of community exercise and chronic disease management. This represents an important gap in the literature, particularly given the increasing emphasis on evidence-based service design and integrated care.

Despite a substantial evidence base supporting exercise for chronic disease management, translating this evidence into routine healthcare practice remains a major implementation challenge (7). Healthcare systems require scalable, evidence-informed service models capable of integrating clinical rehabilitation with community-based physical activity. Community exercise programmes and intermediary coordination roles represent applied solutions that bridge this evidence-practice gap and support sustainable models of integrated care.

Therefore, this review aims to synthesise the latest evidence on community exercise programmes for chronic disease and to examine the emerging PAfHO role in Ireland. In doing so, it seeks to situate the PAfHO model within the international evidence base and explore its potential implications for clinical outcomes, service delivery, and health system performance.

## 2 Methods

### 2.1 Review design

A narrative review was undertaken to examine contemporary evidence relating to community-based exercise programmes for people living with chronic disease and to consider this evidence in the context of the emerging Physical Activity for Health Officer (PAfHO) model in Ireland. The review was intended to provide a broad synthesis of evidence across clinical, community, health-service and policy contexts rather than to undertake a formal systematic review or meta-analysis.

### 2.2 Search strategy and information sources

Electronic searches were conducted in PubMed, Scopus and Web of Science for literature published from January 2020 to 2026. The final database search was conducted on 30th June 2026. Search terms were developed around four principal concepts: (1) physical activity and exercise; (2) chronic disease; (3) community-based provision and exercise referral; and (4) integrated healthcare-community pathways. Search terms included combinations of keywords and Boolean operators such as physical activity, exercise, community exercise, community-based exercise, exercise referral, exercise prescription, chronic disease, chronic condition, long-term condition, integrated care, social prescribing, signposting, healthcare-community partnership, and physical activity pathway.

Additional targeted searches were undertaken to identify literature relating specifically to the development of Physical Activity for Health Officers in Ireland. Because the PAfHO initiative is relatively recent and has generated limited peer-reviewed literature, relevant grey literature was also considered. This included national policy and strategy documents, government and health-service publications, Sport Ireland and Local Sports Partnership documentation, programme reports, and other publicly available service-development material relevant to the implementation of community physical activity pathways in Ireland.

Where a well-established international model required historical context, seminal sources published before 2020 were used for description only and were not treated as part of the contemporary 2020–2026 evidence synthesis.

### 2.3 Eligibility criteria and literature selection

Titles and abstracts were initially screened for relevance to the review objectives. Sources were considered eligible where they addressed community-based exercise or physical activity for adults with chronic disease, exercise referral or signposting pathways, healthcare-community integration, implementation of community physical activity services, or the development and delivery of the PAfHO initiative. Sources focusing exclusively on competitive sport, paediatric populations, acute rehabilitation, or interventions delivered solely within hospital settings without a community transition component were excluded.

Following initial screening, potentially relevant full texts were reviewed, and sources were selected based on their relevance to the review questions, contemporary applicability and contribution to understanding either the effectiveness of community exercise interventions or the organisation and implementation of integrated physical activity pathways. Reference lists of key reviews and policy documents were also examined to identify additional relevant sources.

The searches identified 1,116 (Figure 1) primary/secondary records (Pubmed (*n* = 428), Scopus (*n* = 365), Web of Science (*n* = 323) of which 135 were retained following title and abstract screening with 35 primary/secondary sources included in the final narrative synthesis. Of the 105 reports excluded following review, 29 were removed because they were not entirely community-based interventions, 24 involved the wrong population, including participants under 18 years of age or individuals without a chronic disease, and 22 were excluded because they were not intervention-based studies. A further 18 reports were removed due to insufficient outcome data, while 12 studies were excluded because they were not published in English or were otherwise inaccessible for review. A total of 45 records were identified from grey literature sources, comprising 18 reports, 12 policy documents, and 15 additional grey literature publications. A small number (*n* = 5) of these sources were included to capture relevant evidence and practice-based information that may not have been available through peer-reviewed academic databases, thereby providing a broader understanding of the topic and supporting a more comprehensive review. Excluded documents lacked substance and true relevancy towards the review.

**Figure 1:**
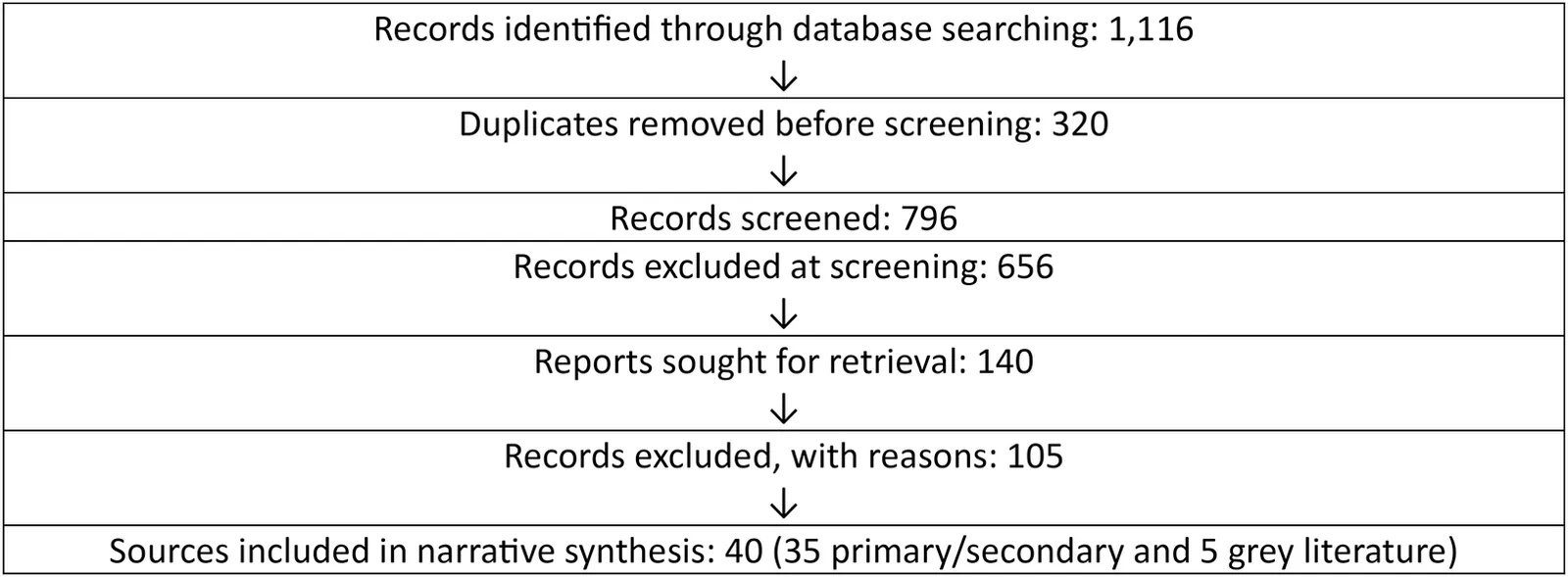
Literature identification and selection process.

The final evidence base comprised systematic and scoping reviews, primary research studies, policy and strategy documents, and relevant grey literature. Because of the heterogeneity of study designs, populations, interventions and outcome measures, findings were synthesised narratively rather than quantitatively.

The evidence was interpreted according to source type. Greater weight was placed on systematic reviews, meta-analyses and peer-reviewed primary studies when considering intervention effectiveness. Policy documents, implementation reports and local service evaluations were used principally to describe health-system context, service development and emerging implementation models rather than as evidence of intervention effectiveness.

## 3 Results

The literature reviewed a diverse range of evidence sources, including systematic reviews and meta-analyses, primary studies, policy documents, service evaluations, and implementation reports. These source types were not treated as equivalent: peer-reviewed reviews and primary studies were used principally to assess intervention effectiveness, whereas policy and service documents were used to describe system context, implementation and the emerging PAfHO model.

Table 1 provides a summary of the key themes identified within the literature, the principal findings associated with each theme, and the studies and reports that informed the narrative synthesis.

**Table 1:** Includes 40 sources of relevant studies selected to match the review scope.

	Authors, Year	Setting	Population and study design	Key outcomes reported
1	Alcazar et al., 2020 (8)	Copenhagen Sarcopenia Study, Denmark	Community-dwelling older adults (60 to 93 years); cross-sectional validation study	30-s sit-to-stand (STS) power strongly correlated with leg extension power and was more strongly associated with gait speed than strength measures, supporting STS power as a valid indicator of functional performance.
2	Alexander et al., 2025 (9)	Community MSK Physiotherapy Service, NHS Lanarkshire	Patients on physiotherapy waiting lists; mixed-methods service evaluation	Community Appointment Days reduced waiting times, increased service capacity, enabled same-day discharge for 12% of patients, and achieved high patient satisfaction.
3	Barakou et al., 2023 (10)	International healthcare and community settings	Adults with chronic conditions from 38 RCTs; systematic review and meta-analysis	Physical activity interventions produced moderate reductions in fatigue, with resistance, aerobic cycling and combined exercise programmes showing the greatest effects.
4	Bull et al., 2020 (1)	Global/public health	General population; WHO guideline	Updated physical activity and sedentary behaviour recommendations across the life course; reinforced that any activity is better than none.
5	Collado-Mateo et al., 2021 (6)	International evidence from chronic disease and ageing populations	Older adults and people with chronic diseases; umbrella review (55 reviews)	Identified 14 key exercise adherence factors including supervision, social support, self-efficacy, education, goal setting, technology, feedback and programme design.
6	Coventry et al., 2021 (11)	Community/nature-based settings	Adults in nature-based interventions; systematic review and meta-analysis	Gardening, green exercise and nature-based therapy improved mental health outcomes and supported wellbeing.
7	de Amorim et al., 2025 (12)	Rehabilitation settings	People with musculoskeletal conditions; systematic review	Common barriers included time, pain and health status; key facilitators included self-efficacy and social support.
8	Dempsey et al., 2020 (13)	Global guideline commentary	General population; policy/guideline commentary	Highlighted the importance of reducing sedentary behaviour and integrating sedentary time guidance with physical activity advice.
9	Department of Health, 2025 (40)	Ireland/national policy	National policy framework and action plan	Set strategic national actions for physical activity, prevention and cross-sector delivery to 2040/2029.
10	Dibben et al., 2023 (14)	Cardiac rehabilitation	Adults with coronary heart disease; Cochrane review	Exercise-based cardiac rehabilitation reduced cardiovascular mortality, hospital admissions and improved quality of life.
11	Dommershuijsen et al., 2022 (15)	Rotterdam Study, Netherlands	Community-dwelling adults aged 50 + years; cross-sectional population study	Established normative gait speed values. Gait speed declined with age and was influenced by height but not sex after adjustment.
12	Elley et al., 2003 (16)	42 general practices, New Zealand	Sedentary adults aged 40 to 79 years; cluster randomised controlled trial	Physical activity counselling increased activity levels, leisure exercise, and quality of life over 12 months.
13	Faíl et al., 2022 (17)	Community/aquatic settings	Adults with and without chronic disease; systematic review and meta-analysis	Aquatic exercise improved health and physical fitness, with benefits evident after 8 to 16 weeks for chronic conditions.
14	Fairag et al., 2024 (18)	Multiple chronic disease management settings	People with cardiovascular disease, diabetes, obesity and mental health conditions; comprehensive review	Exercise reduced mortality risk, improved disease outcomes and quality of life, and was identified as a cost-effective therapeutic intervention.
15	Hayden et al., 2021 (19)	Primary care/rehabilitation	Adults with chronic low back pain; Cochrane review	Exercise was probably effective for pain and function compared with no treatment, usual care or placebo.
16	HSE, 2021 (20)	Ireland/community rehabilitation	Adults requiring community rehabilitation; model of care	Supported person-centred, multidisciplinary, community-based rehabilitation closer to home.
17	Husk et al., 2020 (21)	Primary care/community interface	Social prescribing interventions; realist review	Social prescribing can help people with long-term conditions when local infrastructure and personalised support are in place.
18	Inkpen et al., 2024 (22)	Exercise referral schemes involving healthcare and exercise professionals	Adults; systematic review and meta-analysis	Exercise referral schemes significantly improved cardiorespiratory endurance and fitness outcomes.
19	Izquierdo et al., 2025 (23)	Global consensus/ageing	Older adults; expert consensus	Recommended multicomponent exercise combining aerobic, resistance, balance and flexibility for healthy ageing and chronic disease prevention.
20	Jørgensen et al., 2024 (24)	Orthopaedic and rehabilitation settings, Denmark	Patients with advanced knee osteoarthritis; cross-sectional study	STS power strongly predicted functional performance and patient-reported outcomes and outperformed knee extensor strength as a predictor.
21	Lanzi et al., 2023 (25)	Vascular rehabilitation setting	Patients with peripheral artery disease; observational longitudinal study	STS power was associated with walking performance, gait speed, quality of life and improvements following supervised exercise training.
22	Longford Sports Partnership, 2025 (26)	Ireland/local service implementation	PAfH service description	Outlined local programmes for older adults and people with chronic conditions, focusing on access and progression.
23	Mino et al., 2023 (3)	International/referral schemes	Physical activity referral schemes; systematic review and narrative synthesis	Identified 19 core components of referral schemes and highlighted major heterogeneity in delivery, reporting and follow-up.
24	Mino et al., 2024 (4)	Physical Activity Referral Schemes (PARS) internationally	Adults referred through healthcare settings; systematic review, meta-analysis and meta-regression (52 studies)	PARS increased physical activity compared with usual care. Person-centred approaches, screening and brief advice improved adherence rates.
25	Nøhr et al., 2025 (27)	Digital/chronic disease management	Adults with chronic conditions; systematic review and component network meta-analysis	Exercise combined with self-management, delivered by mixed technologies or web/app platforms, most effectively increased physical activity.
26	O’Brien et al., 2025 (2)	International/healthcare referral systems	Patients in exercise referral schemes; umbrella review	Low-to-moderate improvements in physical activity, fitness and selected health outcomes; cost-effectiveness evidence remained mixed.
27	Park & Shin, 2024 (28)	Geriatric and sarcopenia assessment settings	Older adults at risk of sarcopenia; narrative review	Confirmed the value of the STS test for assessing lower limb strength and function and highlighted its role in sarcopenia screening.
28	Rhodes et al., 2021 (29)	Physical activity behaviour-change interventions	Adult populations; systematic review and meta-analysis	Behaviour change interventions had small but significant mediation effects; no single behaviour-change theory dominated.
29	Rossiter et al., 2025 (5)	International/referral networks	Healthcare professionals and referral stakeholders; review	Exercise referral works best as a collaborative network with feedback loops, shared responsibility and local partnerships.
30	Rutherford et al., 2025	Home-based pulmonary rehabilitation	Adults with COPD; qualitative study	Participants valued accessibility and flexibility of home-based rehabilitation, while motivation and support influenced engagement.
31	Saz-Lara et al., 2024 (7)	Primary care	Healthcare professionals and patients with chronic diseases; evidence-practice implementation study/protocol	Highlighted the gap between exercise evidence and clinical practice and aimed to develop guidelines to support routine exercise prescription.
32	Sharp et al., 2026 (30)	Exercise Referral Scheme, Manchester	Adults referred to ERS; retrospective cohort study	Adherence was influenced by age, deprivation, referral pathway and early attendance. Early engagement was the strongest predictor of programme completion.
33	Sheill et al., 2023 (31)	Hospital-to-community referral pathway, Ireland	Adults with chronic conditions (mean age 72 years); pilot mixed-methods study	Demonstrated feasibility of a hospital-to-community exercise referral pathway, with high satisfaction and 78% intending to maintain membership.
34	Skou et al., 2025 (32)	Multicentre clinical/community care	Adults with multimorbidity; pragmatic assessor-blinded RCT	Personalised exercise plus self-management improved health-related quality of life at 12 months without compromising safety.
35	Smith et al., 2021 (33)	Primary care and community settings	Adults with multimorbidity; systematic review	Targeted interventions can improve selected health outcomes in multimorbidity, especially when addressing function and mental health.
36	Tierney et al., 2024 (34)	Social prescribing services across England	Patients referred to link workers; realist evaluation	Trust, personalised support, patient readiness and strong relationships with link workers were key drivers of engagement and participation.
37	Valenzuela et al., 2023 (35)	International/community and residential care	Older adults; systematic review and meta-analysis	Exercise improved overall physical function, mobility, lower-limb strength and balance in older adults.
38	Wexford County Council, 2025 (36)	Ireland/local service implementation	PAfHO service description	Outlined PAfHO support for people with chronic conditions to re-engage with physical activity.
39	WHO, 2022 (37)	Global policy monitoring	Global population; status report	Reported widespread physical inactivity and uneven implementation of physical activity policy internationally.
40	Wilson et al., 2025 (38)	Community/voluntary sector/primary care	People with long-term health conditions; review	Social prescribing linked people to community supports, with implementation success dependent on local capacity and navigation support.

### 3.1 Evidence for community-based exercise in chronic disease

The evidence reviewed consistently supports the role of community exercise programmes as an important component of chronic disease management. Across a range of chronic conditions, participation in structured exercise programmes is associated with improvements in physical function, physical activity participation, health-related quality of life, and psychological wellbeing (2, 18). These findings align with international recommendations that position physical activity as a cornerstone intervention for the prevention and management of chronic disease (1, 13).

A recurring theme within the literature is that programme effectiveness is influenced not only by the exercise intervention itself but also by the broader context in which it is delivered. Community-based programmes that incorporate personalised exercise prescription, behavioural support, and ongoing participant engagement tend to demonstrate greater improvements in health outcomes than programmes relying solely on referral or information provision (3, 5). This reflects a growing recognition that sustainable behaviour change requires more than access to exercise opportunities alone and must address motivational, social, and environmental determinants of participation.

The benefits of community exercise programmes extend beyond increases in physical activity. Evidence from diverse populations indicates improvements in functional capacity, including mobility, balance, lower limb strength, and walking ability, all of which are closely associated with independence and quality of life (24, 28, 35). Functional measures such as gait speed and sit-to-stand performance have emerged as clinically meaningful indicators of health status and are increasingly used to evaluate programme effectiveness within rehabilitation and community settings (15, 25).

The literature also highlights the value of exercise across a broad range of chronic disease populations. Positive outcomes have been reported in individuals with cardiovascular disease, musculoskeletal disorders, multimorbidity, and long-term health conditions, reinforcing the versatility of exercise as a therapeutic intervention (14, 19, 32). Furthermore, alternative approaches such as aquatic exercise and nature-based physical activity have demonstrated benefits for both physical and mental health outcomes, suggesting that diverse programme formats may enhance accessibility and engagement for different populations (11, 17).

Despite these benefits, the literature demonstrates considerable variability in programme outcomes. Differences in intervention intensity, supervision, duration, behavioural support, and participant characteristics contribute to heterogeneous findings across studies (2, 4). Adherence remains a persistent challenge, with participation influenced by individual, social, and environmental factors. Common barriers include pain, poor health, time constraints, and limited access to suitable programmes, whereas social support, self-efficacy, perceived health benefits, and positive previous experiences facilitate engagement (6, 12).

Beyond individual-level outcomes, community exercise programmes may offer broader benefits for healthcare systems. Emerging evidence suggests that integrated community pathways can support transitions from clinical rehabilitation to long-term self-management, potentially improving service efficiency and reducing pressure on rehabilitation and physiotherapy services (9, 31, 39). Although further evaluation is required, these findings support the growing policy emphasis on community-based models of care and prevention.

Overall, the literature suggests that community exercise programmes represent an effective and scalable approach to chronic disease management. Their impact is greatest when programmes are personalised, supported by behaviour change strategies, and integrated within wider health and community systems. As healthcare systems increasingly prioritise prevention, self-management, and care closer to home, community exercise programmes are likely to play an increasingly important role in supporting population health and reducing demand on clinical services.

### 3.2 Exercise referral, behavioural support and long-term adherence

The literature consistently demonstrates that the effectiveness of community exercise programmes is influenced not only by the exercise intervention itself but also by the design and implementation of referral pathways. Across a range of settings, programme success appears to be strongly associated with personalisation, behavioural support, and structured follow-up, all of which contribute to improved adherence and clinical outcomes (3). Interventions that tailor exercise to individual needs, preferences, and health status, while incorporating behaviour change techniques such as goal setting, feedback, self-monitoring, and social support, are more likely to facilitate sustained engagement and long-term participation in physical activity.

A recurring theme within the literature is the challenge of maintaining participation beyond initial programme enrolment. Although exercise referral schemes are generally associated with small-to-moderate improvements in physical activity participation, fitness, and selected health outcomes, adherence and long-term maintenance of behaviour change remain persistent challenges (2, 4, 30). Evidence suggests that participant engagement is influenced by a complex interaction of demographic, socioeconomic, behavioural, and service-level factors. Variables such as age, socioeconomic deprivation, referral pathway, and early attendance have been identified as important predictors of programme completion and ongoing participation (30).

The literature increasingly emphasises that referral pathways should not be viewed as isolated mechanisms but rather as components of broader systems of care. Effective pathways require collaboration between healthcare professionals, exercise providers, community organisations, and programme coordinators, supported by strong communication processes and feedback mechanisms (5). In this context, referral schemes appear most effective when embedded within integrated systems that provide ongoing support, clear eligibility criteria, coordinated communication between stakeholders, and opportunities for progression into sustainable community-based physical activity (5). These findings suggest that referral alone is unlikely to produce meaningful long-term behaviour change without complementary strategies that address motivation, accessibility, and ongoing engagement.

Within contemporary models of integrated care, there has been increasing recognition of the value of signposting as a complementary or alternative approach to traditional referral. While referral often implies a formal transfer of responsibility between services, signposting reflects a more flexible and person-centred process whereby individuals are supported to identify and access appropriate community opportunities. This distinction may be particularly relevant within physical activity promotion, where sustained participation depends not only on clinical recommendation but also on individual choice, motivation, perceived relevance, and local accessibility.

The concept of signposting aligns closely with current approaches to self-management support and shared decision-making, both of which emphasise individual empowerment and active participation in health behaviours. In practice, many community exercise pathways rely less on formal referral mechanisms and more on navigation, encouragement, and support to overcome barriers to participation. Consequently, the role of healthcare professionals and intermediary roles such as Physical Activity for Health Officers extends beyond referral to encompass facilitation, coordination, advocacy, and behaviour change support.

The literature further suggests that successful referral and signposting pathways are underpinned by broader system-level supports. Accessible programmes, appropriately trained instructors, clear communication channels between services, and mechanisms for monitoring engagement and outcomes all contribute to pathway effectiveness. In the absence of these supports, pathways risk becoming fragmented, limiting programme uptake, reducing adherence, and compromising sustainability.

Overall, the evidence indicates that while exercise referral schemes remain an important strategy for promoting physical activity among individuals with chronic disease, their effectiveness is enhanced when they are situated within integrated systems of care that prioritise person-centred support, navigation, and long-term engagement. A broader conceptualisation that incorporates referral, signposting, and ongoing behaviour change support may therefore provide a more accurate reflection of best practice in community-based physical activity promotion and chronic disease management (2–5, 30).

### 3.3 Integrated healthcare-community models

The literature suggests that community exercise programmes have the potential to contribute to health system performance through improved access to physical activity, enhanced self-management, and more efficient use of healthcare resources. As healthcare systems increasingly face the challenges associated with population ageing, rising rates of chronic disease, and workforce constraints, community-based physical activity programmes are being recognised as an important component of integrated models of care (37, 39).

A recurring theme across the literature is the role of community exercise programmes in supporting prevention and long-term condition management. By providing structured and accessible opportunities for physical activity outside traditional healthcare settings, these programmes can facilitate sustained engagement in health-promoting behaviours and support individuals in managing chronic conditions more independently (1, 18). This emphasis on self-management aligns closely with contemporary healthcare policy, including Sláintecare, which advocates for prevention, early intervention, and care delivered closer to home (39).

The evidence also highlights the potential of community exercise programmes to act as an extension of rehabilitation services. Several studies have identified the value of structured pathways linking healthcare services with community-based physical activity opportunities, particularly for individuals transitioning from formal rehabilitation to long-term self-management (5, 31). Such pathways may help maintain gains achieved during clinical intervention while providing ongoing support in a less resource-intensive setting. This is particularly relevant in physiotherapy and rehabilitation services, where increasing demand often exceeds available capacity.

From a service delivery perspective, community exercise programmes may offer a more scalable approach than traditional one-to-one clinical interventions. Group-based models allow larger numbers of individuals to access supervised exercise simultaneously, potentially improving efficiency while maintaining support and oversight (2). Emerging service evaluations and system-level analyses suggest that integrated community pathways may contribute to improved service flow, reduced reliance on individual clinical appointments, and reductions in waiting list pressures when implemented within coordinated care systems (9, 31, 39). However, the evidence remains limited, and findings are often influenced by local context, programme design, referral processes, and resource availability.

The literature further emphasises the importance of coordination and system integration in maximising the impact of community exercise programmes. Effective pathways require collaboration between healthcare providers, community organisations, exercise professionals, and programme coordinators to ensure continuity of care and appropriate participant support (5). Within the Irish context, this has particular relevance for the Physical Activity for Health Officer (PAfHO) model, which seeks to strengthen connections between HSE services and community provision through signposting, partnership development, and pathway coordination. Such intermediary roles may help address fragmentation between sectors and facilitate more efficient utilisation of existing resources.

Despite these potential benefits, evidence relating to healthcare utilisation and cost-effectiveness remains less conclusive. While some studies report reductions in healthcare use and improvements in service efficiency, others demonstrate more variable outcomes, reflecting differences in intervention design, implementation fidelity, participant characteristics, and local infrastructure (2, 5). Furthermore, much of the available evidence focuses on short-term outcomes, limiting understanding of long-term sustainability and economic impact.

Overall, the literature suggests that community exercise programmes represent a promising strategy for supporting integrated care, enhancing self-management, and improving service efficiency. Their potential contribution to health system performance appears greatest when programmes are embedded within coordinated pathways that facilitate transitions between healthcare and community settings. However, further research is required to establish their long-term impact on healthcare utilisation, cost-effectiveness, and population health outcomes, particularly within the Irish healthcare system.

### 3.4 Irish policy context and development of the PAfHO role

An important distinction should be made between the evidence supporting community-based exercise interventions and the evidence directly evaluating the PAfHO model. Community exercise, exercise referral and integrated physical activity interventions are supported by an established international literature that includes systematic reviews, controlled studies and implementation research. In contrast, the PAfHO initiative represents a relatively recent development within the Irish system, and direct empirical evaluation of the role remains limited.

Consequently, current evidence relating specifically to PAfHOs is derived predominantly from policy documentation, programme descriptions, implementation experience and emerging service-level evaluations rather than comparative effectiveness studies. These sources are valuable for describing the rationale, structure and potential functions of the role but should not be interpreted as providing the same level of evidence as peer-reviewed intervention studies demonstrating clinical effectiveness.

The growing emphasis on prevention, self-management, and integrated care has led to increased recognition of the need for intermediary roles that can bridge healthcare and community-based physical activity provision. Within Ireland, the Physical Activity for Health Officer (PAfHO) pilot represents an emerging approach to addressing this challenge. Positioned across six counties and operating at the interface of the Health Service Executive (HSE), Sport Ireland, and local authorities/sports partnerships, the role seeks to strengthen pathways between clinical services and community-based physical activity opportunities through coordination, programme development, and stakeholder engagement.

The literature reviewed suggests that effective community physical activity pathways require more than the availability of exercise programmes alone. Access, navigation, continuity of care, and sustained participant support have emerged as critical components of successful systems (2, 5). In this context, the PAfHO model can be viewed as a response to a recognised implementation gap between healthcare services and long-term participation in physical activity. Emerging Irish evidence indicates that the role may facilitate access to structured and inclusive physical activity opportunities while supporting transitions from rehabilitation services to community-based self-management. However, current evidence is derived primarily from local service reports and practice-based evaluations and therefore remains exploratory rather than definitive.

Internationally, the PAfHO model shares similarities with a number of intermediary and navigation-based approaches that have emerged within integrated care systems. Social prescribing link workers in England provide one example, supporting individuals to identify non-medical factors affecting their health and connecting them with appropriate community resources through a person-centred approach (34). While social prescribing encompasses a broader range of social and health needs than physical activity alone, both models emphasise navigation, partnership working, and individualised support. Nevertheless, evaluations of social prescribing have demonstrated considerable variability in outcomes, highlighting the importance of implementation fidelity, local infrastructure, workforce capacity, and service integration in determining programme success (34).

Similarly, the Green Prescription model in Aotearoa New Zealand provides a structured mechanism through which healthcare professionals can support physical activity behaviour change via written advice, counselling and follow-up support (16). Trial evidence from the model has demonstrated increases in physical activity and improvements in selected quality-of-life outcomes, although long-term maintenance and the transferability of findings to other health systems remain important considerations (2, 16).

The broader exercise referral literature further supports the importance of system-level integration. Recent reviews indicate that referral mechanisms alone are insufficient to produce sustained changes in physical activity behaviour and that effectiveness is enhanced when pathways incorporate behavioural support, stakeholder collaboration, and coordinated participant follow-up (2, 5). Viewed through this lens, the PAfHO model appears conceptually aligned with current evidence, as its core functions extend beyond programme delivery to include facilitation, partnership development, and pathway coordination. These characteristics position the role within a broader movement towards integrated and community-oriented models of care.

Despite its strong alignment with national policy priorities, including Sláintecare and the National Physical Activity Framework, the evidence base underpinning the PAfHO model remains limited. Important questions remain regarding its impact on physical activity participation, functional outcomes, healthcare utilisation, service efficiency, and cost-effectiveness. While existing reports suggest positive contributions to pathway development and community engagement, robust outcome evaluation is required before definitive conclusions can be drawn regarding effectiveness or scalability.

## 4 Discussion and future directions

The literature reviewed highlights the growing importance of community exercise programmes within contemporary approaches to chronic disease management. Across diverse populations and settings, the evidence suggests that programmes are most effective when delivered as part of integrated care pathways rather than as isolated interventions. A consistent theme throughout the literature is the importance of combining structured exercise opportunities with behavioural support, participant engagement strategies, and coordinated pathways between healthcare and community services. Such approaches align with broader healthcare priorities that emphasise prevention, continuity of care, self-management, and care delivered closer to home.

From a health systems perspective, community exercise programmes are increasingly viewed as a means of extending care beyond traditional clinical settings. The literature suggests that these programmes may contribute to more efficient use of healthcare resources by providing opportunities for individuals to continue physical activity and rehabilitation within community settings following discharge from formal healthcare services. This has relevance for healthcare systems experiencing increasing demand for physiotherapy, rehabilitation, and chronic disease management services. While emerging evidence indicates that community pathways may support service efficiency and improve transitions between services, the extent to which these programmes influence healthcare utilisation, waiting list management, and workforce capacity remains an area requiring further investigation.

The evidence reviewed also highlights the importance of intermediary roles that facilitate navigation between healthcare and community provision. Within Ireland, the Physical Activity for Health Officer (PAfHO) model represents one such approach. The role is conceptually aligned with international evidence supporting coordinated referral pathways, behavioural support, and cross-sector collaboration. By facilitating signposting, programme development, stakeholder engagement, and pathway coordination, the PAfHO model seeks to address a commonly reported challenge within the literature: sustaining participation in physical activity following completion of clinical rehabilitation or structured healthcare interventions. The transition from supervised rehabilitation to independent community participation is frequently identified as a vulnerable period during which individuals may disengage from physical activity, potentially leading to declines in function and health status.

The literature further demonstrates that community exercise programmes can produce improvements in clinically meaningful outcomes, including physical function, mobility, balance, gait speed, muscular strength, and self-reported wellbeing. These outcomes are particularly relevant for individuals living with chronic disease, disability, and multimorbidity, where even modest improvements in functional capacity may contribute to enhanced independence, reduced fall risk, and improved quality of life. However, considerable heterogeneity exists across studies, reflecting differences in programme design, participant populations, behavioural support components, and implementation contexts.

Despite the apparent alignment between the PAfHO model and the broader evidence base, a notable gap remains regarding formal evaluation of the role itself. Much of the available evidence relating to PAfHO implementation is currently derived from grey literature, local programme reports, and service evaluations. While these sources provide valuable insight into programme delivery and perceived impact, they offer limited evidence regarding effectiveness, scalability, and cost-effectiveness. This reflects a broader challenge within community-based physical activity research, where implementation frequently precedes rigorous evaluation.

Taken together, the findings of this review suggest that the key challenge facing health systems is not whether community exercise programmes can improve health outcomes, but how such programmes can be implemented, coordinated, and sustained at scale. The literature increasingly supports a shift away from viewing physical activity solely as a clinical recommendation and towards recognising it as an integrated component of healthcare delivery. In this context, intermediary roles such as the PAfHO may represent an important mechanism for translating policy aspirations into operational practice.

Future research should prioritise robust evaluation of the PAfHO model and similar pathway-based approaches. Attention should be given to clinical outcomes, physical activity participation, quality of life, and long-term behaviour change, alongside system-level indicators such as healthcare utilisation, waiting list management, service efficiency, and cost-effectiveness. Comparative studies across regions with differing levels of pathway integration would provide valuable insight into the added value of intermediary roles. In addition, implementation-focused research is required to better understand the contextual factors that influence programme success, including workforce capacity, referral processes, stakeholder engagement, rurality, accessibility, and programme fidelity.

The findings of this review have practical implications for healthcare providers, commissioners, policymakers, and community organisations seeking to develop sustainable physical activity pathways. Rather than viewing exercise programmes as isolated interventions, evidence suggests that they function most effectively when embedded within integrated service models supported by coordinated referral pathways and dedicated implementation roles.

The emerging Physical Activity for Health Officer model provides an example of how implementation science principles can be operationalised within routine practice. By coordinating stakeholders across healthcare, local government, and community organisations, the role addresses common barriers to implementation including fragmented referral pathways, poor communication between sectors, and limited continuity following discharge from rehabilitation.

From an applied perspective, this model demonstrates how existing community resources can be integrated to improve access to evidence-based exercise while potentially reducing demand on healthcare services. Future implementation should include standardised referral/signposting criteria, shared outcome measurement, digital referral/signposting systems, workforce training, and continuous service evaluation to maximise scalability and sustainability.

Evidence directly evaluating the Physical Activity for Health Officer (PAfHO) model remains at an early stage. In contrast to the established evidence base supporting community-based exercise and physical activity interventions for people living with chronic disease, current evidence relating specifically to the PAfHO role is derived largely from policy documents, programme descriptions, implementation experience and emerging service-level evaluations. Consequently, the potential contribution of the role should be distinguished from outcomes that have been demonstrated through controlled or comparative research.

The PAfHO model has the potential to address recognised gaps between healthcare and community-based physical activity provision by supporting referral and signposting pathways, identifying appropriate local opportunities, and facilitating collaboration between healthcare professionals, Local Sports Partnerships and community providers. These functions are consistent with wider evidence supporting integrated approaches to physical activity promotion and the importance of maintaining physical activity following discharge or transition from healthcare-based services. However, evidence that these potential mechanisms translate into improved participant outcomes or measurable health-service benefits specifically as a result of PAfHO involvement is currently limited.

Several important evidence gaps therefore remain. Prospective evaluation is required to determine whether PAfHO-supported pathways improve referral uptake, access to community programmes, adherence and longer-term physical activity participation compared with existing approaches. Evaluation should also examine clinical and functional outcomes, participant experience, equity of access, programme retention and progression to independent physical activity. At a health-service level, future research should investigate whether the model influences healthcare utilisation, waiting lists, repeat referrals or demand for healthcare-led exercise services.

Economic evaluation is also required to establish the cost-effectiveness and sustainability of the model. As the PAfHO initiative develops, the collection of standardised outcome and implementation data across participating areas would enable comparison between different delivery models and provide stronger evidence regarding effectiveness, scalability and potential integration within the wider Irish health system.

Overall, the literature suggests that community exercise programmes are likely to play an increasingly important role within integrated models of chronic disease management. The PAfHO model represents a promising example of how healthcare and community sectors may be connected to support sustained physical activity participation. However, further evidence is required to determine the extent to which this approach can deliver measurable benefits for individuals, services, and the wider health system.

### 4.1 Limitations

Several limitations should be considered when interpreting this review. First, the narrative review methodology does not provide the same degree of reproducibility or protection against selection bias as a formal systematic review, and relevant studies may therefore have been omitted. Although searches were undertaken across several major electronic databases and supplemented by targeted grey literature searches, study selection was based on relevance to the review objectives rather than a formal systematic-review protocol.

Second, the review was restricted to English-language literature, which may have excluded relevant international evidence. Considerable heterogeneity was also present across the included literature in terms of populations, chronic conditions, intervention models, settings and outcome measures, limiting direct comparison between studies.

Third, grey literature and policy documentation were included because they provide important contextual information regarding the development of integrated physical activity pathways and the PAfHO model in Ireland. However, these sources have not necessarily undergone peer review and should not be interpreted as equivalent to controlled primary research or systematic reviews when considering effectiveness.

Finally, direct empirical evidence evaluating the PAfHO model remains limited. Much of the currently available information relates to programme development, implementation and service delivery rather than controlled evaluation of participant outcomes, healthcare utilisation or cost-effectiveness. Conclusions regarding the potential contribution of PAfHOs should therefore be considered preliminary until prospective evaluations of the model become available.

## 5 Conclusion

This narrative review highlights the growing evidence supporting community exercise programmes as an important component of chronic disease management. Across a range of populations and settings, community-based interventions have demonstrated benefits for physical function, physical activity participation, health-related quality of life, and self-management. The literature further suggests that exercise referral and signposting pathways are most effective when embedded within integrated systems that incorporate behavioural support, coordinated care pathways, and ongoing participant engagement.

Within Ireland, the Physical Activity for Health Officer (PAfHO) model represents a promising development that aligns with national policy priorities focused on prevention, integrated care, and community-based service delivery. By facilitating connections between healthcare services and community physical activity opportunities, the role has the potential to strengthen pathway development and support sustained participation in physical activity among individuals living with chronic disease.

However, despite strong conceptual alignment with international evidence, the PAfHO model remains under-evaluated. Community exercise programmes should be viewed not solely as rehabilitation interventions but as components of integrated health system infrastructure. As healthcare systems increasingly prioritise prevention, population health, and care closer to home, applied models that integrate exercise within routine service delivery are likely to become an increasingly important component of chronic disease management. Future research should prioritise rigorous assessment of clinical outcomes, healthcare utilisation, service efficiency, and cost-effectiveness to determine the long-term value of this emerging model within the Irish healthcare system.
